# Effects of Different Sowing Methods on Winter Rapeseed (*Brassica rapa* L.) Growth and Soil Properties in Saline–Alkali Land

**DOI:** 10.3390/plants15121838

**Published:** 2026-06-14

**Authors:** Hao Sun, Junyan Wu, Yan Fang, Yifan Wang, Zhengnan Xu, Shiyi Li, Yuanyuan Zhang, Lijun Liu, Yuanyuan Pu, Gang Yang, Wangtian Wang, Tingting Fan, Wancang Sun, Li Ma

**Affiliations:** State Key Laboratory of Aridland Crop Science, College of Agronomy, Gansu Agricultural University, Lanzhou 730070, China; 19119910352@163.com (H.S.); wujuny@gsau.edu.cn (J.W.);

**Keywords:** lightly saline–alkaline soil, ridge–furrow precision sowing, winter rapeseed, soil environment, physiological traits, yield

## Abstract

A field experiment was conducted in three ecological zones to evaluate the effects of broadcast sowing (BS), drill sowing (DS), and ridge–furrow precision sowing (RFS) on winter rapeseed (*Brassica rapa* L.) grown in lightly saline–alkaline soils, using two cultivars (L6 and L7). RFS improved soil temperature and soil moisture conditions across the zones. Its warming effect was most pronounced in the JT zone, where soil temperatures at seedling and flowering stages were 9.7% and 10.3% higher than under BS, respectively. RFS also showed a moisture-conservation advantage at regreening, with soil moisture 13.8% and 6.6% higher than under BS and DS, respectively. In addition, RFS reduced soil salinity and increased soil total carbon, available potassium, and ammonium nitrogen contents. Plants under RFS showed higher SPAD values, net photosynthetic rates, and transpiration rates at seedling and regreening stages, along with higher antioxidant enzyme activities and lower MDA accumulation. RFS advanced key phenological stages, improved overwintering survival, and produced the highest yield. Compared with BS and DS, respectively, RFS increased the mean yield of L6 by 11.46% and 6.97%, and that of L7 by 16.02% and 10.52%. Overall, RFS promoted yield formation by improving soil conditions, photosynthetic activity, and stress resistance.

## 1. Introduction

Soil salinization and alkalinization are major constraints on global agricultural production and the sustainable use of land resources. It has been estimated that salt-affected soils account for approximately 10% of the world’s arable land. In China, saline–alkali soils are widely distributed, covering about 36 million ha and accounting for 4.88% of the country’s available land area [1,2]. Soil salinization is particularly severe in the arid and semi-arid regions of northwestern China, where low precipitation and strong evaporation favor salt accumulation in surface soils, thereby worsening the salinization problem [3]. Saline–alkali soils are generally characterized by elevated salinity, high alkalinity, poor soil structure, and low nutrient availability. These unfavorable soil conditions not only inhibit seed germination and root growth, but also impair photosynthesis and disrupt normal plant growth and development, thereby reducing water and nutrient uptake and utilization by crops, ultimately leading to stunted growth and yield loss [4,5]. Therefore, identifying suitable crops and optimizing agronomic practices are important strategies for alleviating salt stress and improving land productivity in saline–alkali regions of northwestern China.

Rapeseed, belonging to the genus Brassica in the family Brassicaceae, is one of the four major oilseed crops worldwide and one of the main sources of edible vegetable oil in China [6,7]. Owing to its relative salt tolerance, rapeseed has considerable potential for the utilization and amelioration of saline–alkali soils [8]. In recent years, with continuous progress in the breeding of cold-tolerant winter rapeseed cultivars, its cultivation area has gradually expanded to the high-latitude and high-altitude regions of northern China. Northwestern China is characterized by abundant solar radiation and large diurnal temperature fluctuations, which provide favorable ecological conditions for the growth, development, and quality formation of winter rapeseed [9,10]. Previous studies have shown that rapeseed can enhance its adaptation to saline–alkali stress by improving antioxidant capacity, promoting the accumulation of osmotic adjustment substances, and regulating endogenous hormone levels [11,12]. In addition, rapeseed cultivation can, to some extent, reduce soil salinity and increase soil organic matter, total nitrogen, and available phosphorus contents, thereby improving soil nutrient status [13,14]. Nevertheless, saline–alkali stress still markedly suppresses photosynthesis and dry matter accumulation in rapeseed, induces excessive reactive oxygen species (ROS) production, aggravates oxidative damage and metabolic disorders, and ultimately results in growth inhibition and yield reduction [15,16,17,18]. It is worth noting that, owing to differences in genetic background, different rapeseed genotypes often exhibit distinct adaptive responses to salt stress, mainly reflected in seedling establishment, growth maintenance, and antioxidant and metabolic regulation, thereby affecting stress adaptation and overall plant performance [19,20]. Therefore, under saline–alkali stress, how to further exploit the production potential of winter rapeseed through cultivation and management practices, while taking cultivar differences into consideration, has become an urgent issue in winter rapeseed production on saline–alkali soils.

Improving the root-zone microenvironment through appropriate agronomic practices is an important approach for enhancing the growth and yield performance of winter rapeseed under saline–alkali conditions. Ridge–furrow precision sowing integrates ridging, furrow opening, and precision sowing, and can regulate the spatiotemporal distribution of soil moisture and salts by modifying surface microtopography. Under rainfall or irrigation conditions, the furrows facilitate water harvesting and promote the downward leaching of salts. During periods of intense evaporation, salts tend to accumulate preferentially in relatively elevated surface areas, thereby creating a relatively low-salinity root-zone microenvironment within the furrows and reducing the risk of salt injury during the seedling stage [21,22,23]. In addition, the ridge–furrow configuration can improve soil aeration as well as soil hydrothermal conditions, thereby promoting root development and plant establishment and laying the foundation for maintaining relatively high photosynthetic productivity and dry matter accumulation [24,25]. However, existing studies have mainly focused on ridge–furrow or mulched ridge–furrow planting systems themselves. It is still unclear whether ridge–furrow precision sowing maintains a stable advantage over traditional broadcasting and drill sowing under saline–alkali conditions. Meanwhile, systematic understanding remains insufficient regarding the coordinated regulatory mechanisms of ridge–furrow precision sowing on plant photosynthesis, antioxidant physiology, growth and development, as well as soil moisture, temperature, and physical properties. In addition, comparative evaluation across different ecological regions remains insufficient.

Therefore, field experiments were conducted in three different lightly saline–alkali ecological regions using three sowing methods, namely broadcasting, drill sowing, and ridge–furrow precision sowing, to systematically evaluate the effects of ridge–furrow precision sowing on the soil environment, physiological characteristics, growth and development, and yield formation of winter rapeseed under lightly saline–alkali conditions. Considering that different rapeseed genotypes may exhibit distinct adaptive responses to salt stress, different winter rapeseed cultivars were also included in this study to test the applicability and stability of the regulatory effects of ridge–furrow precision sowing across different genetic backgrounds. Based on these considerations, we proposed the following hypotheses: (1) ridge–furrow precision sowing would improve the root-zone soil environment by optimizing soil moisture, soil temperature, soil salt content, and related physicochemical properties; (2) ridge–furrow precision sowing would enhance growth, physiological performance, antioxidant defense, and yield formation of winter rapeseed under lightly saline–alkali conditions; and (3) compared with broadcast sowing and drill sowing, both cultivars would perform better under ridge–furrow precision sowing, but the magnitude of improvement would differ between cultivars and ecological zones.

## 2. Results

### 2.1. Effects of Sowing Methods on the Growth Process and Overwintering Rate of Winter Rapeseed in Different Ecological Zones

As shown in Table 1, the total growth period ranged from 302 to 310 days across treatments, indicating limited variation in phenological duration. Among the three sowing methods, broadcast sowing (BS), drill sowing (DS), and ridge–furrow precision sowing (RFS), RFS tended to shorten the overall growth period and promote earlier maturity at most experimental sites. Compared with BS and DS, RFS reduced the total growth period by 2–5 days, with regreening generally occurring 1–3 days earlier. Flowering and maturity were also advanced under RFS, indicating faster post-winter regrowth.

In addition, RFS increased the overwinter survival rate of both cultivars across the three ecological zones, although the magnitude of increase varied among zones. In the SC ecological zone, the overwinter survival rate of L6 under RFS increased by 4.1% and 1.3% relative to BS and DS, respectively, while that of L7 increased by 2.3% and 3.1%, respectively. In the JT ecological zone, the corresponding increases were 7.2% and 3.0% for L6 and 7.7% and 3.6% for L7, indicating the strongest response among the three zones. In the ZY ecological zone, RFS increased the overwinter survival rate by 3.9% and 2.1% for L6 and by 5.7% and 3.9% for L7, respectively, relative to BS and DS. Collectively, RFS increased overwinter survival while maintaining normal phenological development across all three ecological zones.

### 2.2. Effects of Sowing Methods on Soil Temperature and Soil Moisture Conditions in Different Ecological Zones

**Figure 1:**
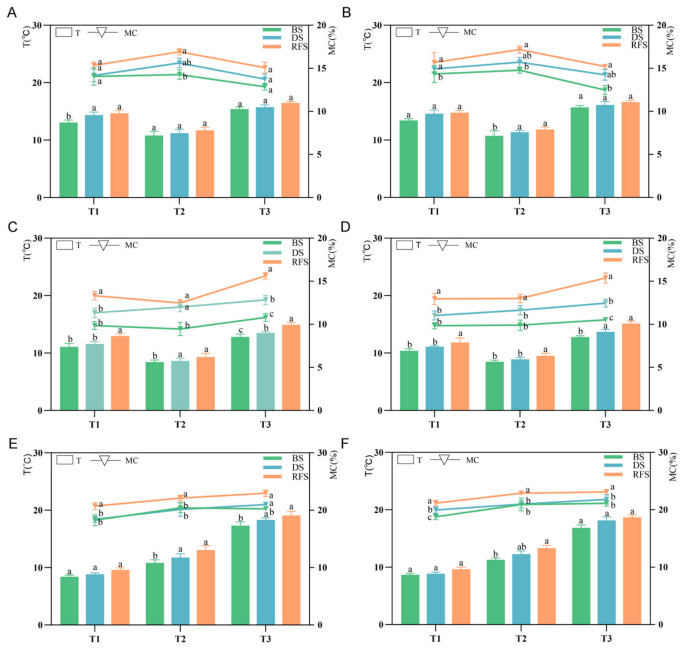
Effects of different sowing methods on soil temperature and soil moisture in winter rapeseed fields at different growth stages across three ecological zones. Bars and lines with inverted triangles represent soil temperature (left *y*-axis, °C) and soil moisture (MC, right *y*-axis, %), respectively. T1, seedling stage; T2, regreening stage; T3, flowering stage. Panels (**A**,**B**), (**C**,**D**), and (**E**,**F**) correspond to the SC, JT, and ZY ecological zones, respectively. Left panels (**A**,**C**,**E**) represent cultivar L6, and right panels (**B**,**D**,**F**) represent cultivar L7. BS, broadcast sowing; DS, drill sowing; RFS, ridge–furrow precision sowing. Values are means ± SE (*n* = 3). Different lowercase letters indicate significant differences among sowing methods within the same growth stage, ecological zone, cultivar, and variable, according to one-way ANOVA followed by Duncan’s multiple range test (*p* < 0.05).

Figure 1 illustrates the dynamic changes in soil temperature and soil moisture in the 0–30 cm soil layer across three ecological zones under different sowing methods, including broadcast sowing (BS), drill sowing (DS), and ridge–furrow sowing (RFS), at the seedling stage (T1), regreening stage (T2), and flowering stage (T3) of winter rapeseed. For soil temperature, the effects of sowing methods showed a generally consistent pattern, following the order RFS > DS > BS. In the JT ecological zone (Figure 1C,D), RFS showed the greatest increase in soil temperature. At T1, soil temperature under RFS was 9.7% and 4.8% higher than that under BS and DS, respectively. At T3, the corresponding increases were 10.3% and 4.0%, respectively. In the SC and ZY ecological zones (Figure 1A,B,E,F), although the differences among treatments were smaller, soil temperature under RFS remained higher than that under BS and DS.

Soil moisture showed a stronger response to sowing method than soil temperature and displayed clear stage-specific variation. The regreening stage was the key period for differentiation in soil moisture among treatments. At T2, soil moisture under RFS was significantly higher than that under BS across all ecological zones. Specifically, soil moisture under RFS was 13.8% and 6.6% higher than that under BS and DS, respectively. Across the observed growth stages, RFS consistently maintained a soil moisture advantage, whereas BS showed the lowest soil moisture. In the SC and ZY ecological zones, soil moisture under RFS remained higher than that under BS at both T1 and T3. Compared with BS, RFS increased soil moisture in SC and ZY by 7.6% and 7.5%, respectively, at T1, and by 10.3% and 7.0%, respectively, at T3.

### 2.3. Effects of Sowing Methods on Soil Physicochemical Properties During the Regreening Period in Different Ecological Zones

According to the results of the three-way ANOVA of soil physicochemical properties at the regreening stage (Table 2), ecological zone (R) had highly significant effects on most soil physicochemical properties (*p* < 0.01) and a significant effect on total phosphorus (TP) (*p* < 0.05), indicating that ecological zone was an important factor affecting variation in soil physicochemical properties. Variety (V) significantly affected total nitrogen (TN), total potassium (TK), total carbon (TC), pH, salt content (Salt), available potassium (AK), available phosphorus (AP), and ammonium nitrogen (NH_4_^+^-N), whereas sowing method (M) significantly affected TN, TK, pH, Salt, AK, SOM, NO_3_^-^-N, and NH_4_^+^-N.

Significant interactions between ecological zone and sowing method were observed for several soil properties, mainly involving total carbon (TC), salt content (Salt), available potassium (AK), and ammonium nitrogen (NH_4_^+^-N). Among them, the R × M interaction for TC was mainly observed in the JT ecological zone, where significant differences were found among sowing methods, and TC under the RFS treatment was significantly higher than that under the BS and DS treatments, with increases of 3.6% and 6.0%, respectively. In contrast, no significant differences among sowing methods were detected in the SC and ZY ecological zones. AK and NH_4_^+^-N both showed differential responses to sowing methods across ecological zones, and the RFS treatment generally maintained higher levels. Specifically, in the SC, JT, and ZY ecological zones, AK under RFS increased by 15.7% and 2.8%, 3.4% and 2.4%, and 9.2% and 1.7% relative to BS and DS, respectively. The corresponding increases in NH_4_^+^-N were 17.2% and 10.0%, 13.8% and 10.1%, and 26.6% and 18.9%, respectively. The response of salt content to sowing method showed clear ecological zone-specific differences. In the SC ecological zone, salt content under the RFS treatment was significantly reduced by 21.69% and 9.72% compared with the BS and DS treatments, respectively. In the JT and ZY ecological zones, no significant differences were detected among treatments; however, overall, salt content under the RFS treatment was lower than that under the BS and DS treatments.

Significant interactions between ecological zone and variety were also detected for multiple soil properties, mainly involving total carbon (TC), pH, salt content (Salt), available potassium (AK), available phosphorus (AP), nitrate nitrogen (NO_3_^-^-N), and ammonium nitrogen (NH_4_^+^-N). TC showed significant cultivar differences among ecological zones. In the JT ecological zone, TC in L7 was significantly higher than that in L6, with an increase of 3.53%; in contrast, in the ZY ecological zone, TC in L6 was significantly higher than that in L7, with an increase of 9.16%. No significant cultivar difference was observed in the SC ecological zone. In the JT and ZY ecological zones, salt content in L7 was significantly lower than that in L6, with reductions of 25.4% and 33.3%, respectively, whereas no significant cultivar difference was found in the SC ecological zone. Meanwhile, in the SC ecological zone, pH in L6 was significantly higher than that in L7 by 0.71%, whereas in the JT and ZY ecological zones, pH in L7 was significantly higher than that in L6 by 1.71% and 1.35%, respectively. Available nutrients and mineral nitrogen also exhibited clear ecological zone-specific cultivar differences. In the SC ecological zone, AP, NO_3_^-^-N, and NH_4_^+^-N in L7 were all significantly higher than those in L6, with increases of 15.9%, 2.1%, and 17.7%, respectively. In the ZY ecological zone, AK in L7 was significantly higher than that in L6, with an increase of 12.0%, whereas no significant cultivar difference in AK was observed in the SC and JT ecological zones. In addition, TN and TK showed significant main effects of ecological zone, variety, and sowing method, whereas the other indicators responded significantly only to some main-effect factors, and their interaction effects were not significant or were relatively weak.

### 2.4. Effects of Sowing Methods on SPAD Values and Photosynthetic Characteristics of Rapeseed in Different Ecological Zones

#### 2.4.1. Effect of Sowing Method on SPAD Values of Rapeseed in Different Ecological Zones

**Figure 2:**
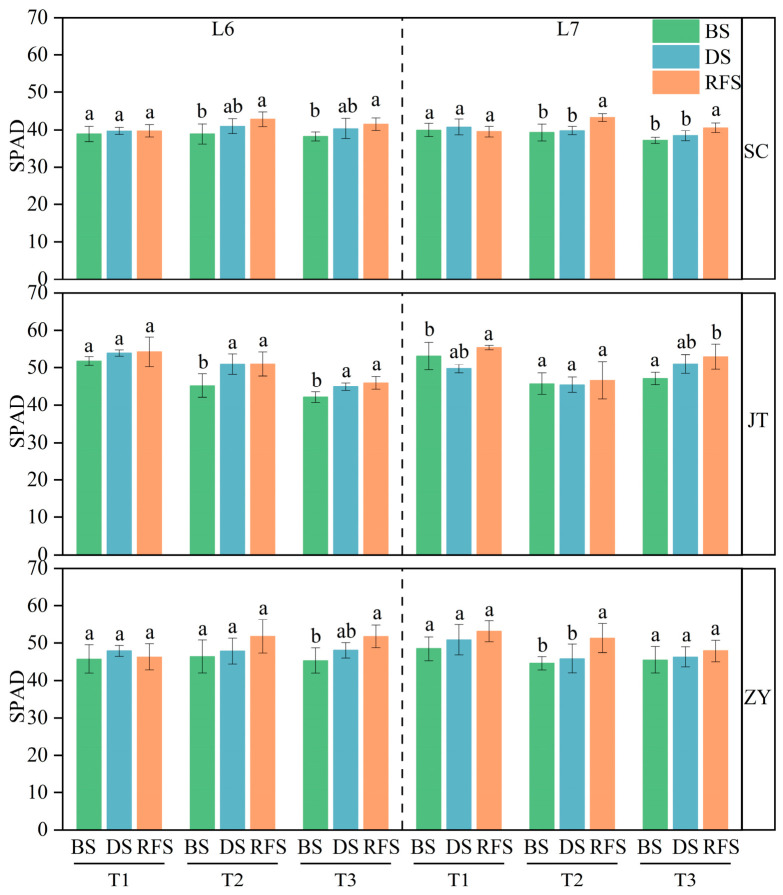
Effects of different sowing methods on leaf SPAD values of winter rapeseed across ecological zones. Rows represent SC, JT, and ZY from top to bottom; the left and right sections separated by the dashed line represent cultivars L6 and L7, respectively. T1, seedling stage; T2, regreening stage; T3, flowering stage. BS, broadcast sowing; DS, drill sowing; RFS, ridge–furrow precision sowing. Values are means ± SE (*n* = 3). Different lowercase letters indicate significant differences among sowing methods within the same ecological zone, cultivar, and growth stage according to Duncan’s multiple range test (*p* < 0.05).

Figure 2 shows the dynamic changes in leaf SPAD values of winter rapeseed across different ecological zones under different sowing methods. Three-way ANOVA indicated that ecological zone, variety, and sowing method all significantly affected leaf SPAD values. In addition, the interaction between ecological zone and variety was significant at both the seedling stage (T1) and flowering stage (T3).

Among the sowing methods, the RFS treatment showed a significant and stable advantage. Across all growth stages, SPAD values under RFS were significantly higher than those under the BS and DS treatments. During T1, SPAD values under RFS were 3.6% and 1.9% higher than those under BS and DS, respectively. During the regreening stage (T2), the corresponding increases were 10.2% and 5.9%, respectively. During T3, SPAD values under RFS remained significantly higher than those under BS and DS, with increases of 9.8% and 4.3%, respectively. Overall, SPAD values under RFS remained higher than those under the BS and DS treatments throughout the entire growth period.

With respect to interaction effects, no significant interaction between ecological zone and variety was detected at T2, and the two factors showed relatively independent main effects at this stage. SPAD values in L6 were significantly higher than those in L7, whereas the JT and ZY ecological zones showed significantly higher SPAD values than the SC ecological zone, by 16.3% and 17.5%, respectively. However, significant interactions between ecological zone and variety were observed at both T1 and T3. Simple-effects analysis showed that, at T1, the SPAD value of L7 in the ZY ecological zone was significantly higher than that of L6 by 8.9%. At T3, the SPAD value of L7 in the JT ecological zone was significantly higher than that of L6 by 16.5%, whereas no significant cultivar differences were observed in the other ecological zones. In summary, ecological zone was an important environmental factor affecting leaf SPAD values, and the RFS treatment maintained relatively high SPAD values across different ecological zones and growth stages.

#### 2.4.2. Effects of Sowing Methods on Photosynthetic Characteristics of Winter Rapeseed in Different Ecological Zones

**Figure 3:**
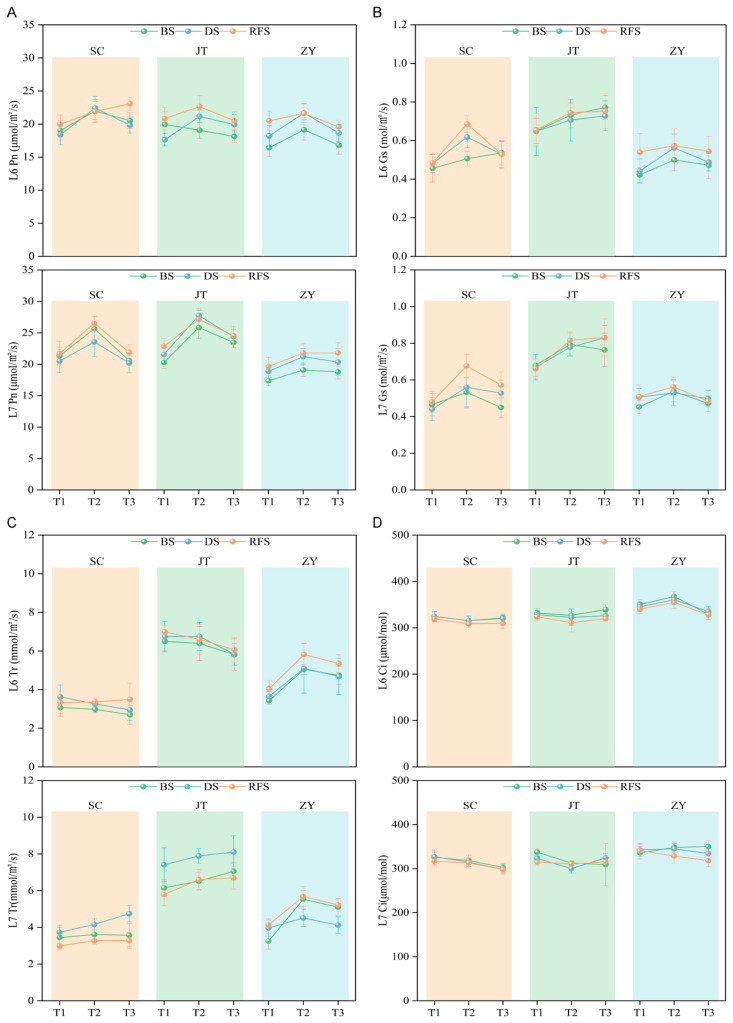
Effects of different sowing methods on photosynthetic characteristics of winter rapeseed at different growth stages across three ecological zones. Panels (**A**–**D**) represent net photosynthetic rate (Pn), stomatal conductance (Gs), intercellular CO_2_ concentration (Ci), and transpiration rate (Tr), respectively. In each panel, the upper and lower subplots represent cultivars Longyou 6 (L6) and Longyou 7 (L7), respectively. The shaded sections from left to right correspond to the SC, JT, and ZY ecological zones, respectively. T1, seedling stage; T2, regreening stage; T3, flowering stage. BS, broadcast sowing; DS, drill sowing; RFS, ridge–furrow precision sowing. Values are means ± SE (*n* = 3).

Figure 3 illustrates the dynamic changes in photosynthetic characteristics under different ecological zones, cultivars, and sowing methods. Pn was significantly affected by ecological zone, cultivar, and sowing method at all three growth stages. Significant interactions between ecological zone and cultivar and between ecological zone and sowing method were detected, whereas the other interactions were not significant. Simple effects analysis showed that the cultivar effect on Pn depended on ecological zone. L7 generally showed significantly higher Pn than L6 in SC and JT at the seedling and regreening stages, with the greatest increase observed in JT at the regreening stage by 22.2%. At the flowering stage, this cultivar advantage was mainly observed in JT and ZY. The effect of the sowing method on Pn also varied among ecological zones. Compared with BS and DS, RFS significantly increased Pn in JT and ZY at the seedling stage, in ZY at the regreening stage, and in SC and ZY at the flowering stage. The largest increase caused by RFS was observed in ZY at the seedling stage, where Pn increased by 15.8% and 7.6% relative to BS and DS, respectively.

Gs and Tr showed different sensitivities to ecological zone, cultivar, and sowing method. For Gs, only the main effect of ecological zone was significant at the seedling and flowering stages, with JT showing significantly higher values than SC and ZY. At the regreening stage, significant interactions between ecological zone and cultivar and between ecological zone and sowing method were detected. Simple effects analysis showed that Gs of L7 was significantly higher than that of L6 only in JT, with an increase of 8.7%. In addition, RFS significantly increased Gs in SC by 23.8% and 13.5% relative to BS and DS, respectively.

Compared with Gs, Tr showed a more consistent response to sowing method. At the seedling stage, Tr was significantly affected by ecological zone and sowing method, whereas the other interactions were not significant. Tr in JT was significantly higher than that in SC and ZY. RFS and DS were both significantly higher than BS, although they did not differ significantly from each other. At the regreening stage, the main effect of the sowing method was significant, with RFS increasing Tr by 9.4% and 7.7% relative to DS and BS, respectively. The interaction between ecological zone and cultivar was also significant, with L7 showing higher Tr than L6 in SC and JT. At the flowering stage, Tr was significantly affected by ecological zone and sowing method, whereas the interactions were not significant. JT showed significantly higher Tr than the other two ecological zones, and RFS increased Tr by 9.9% and 8.9% relative to BS and DS, respectively.

Ci was mainly affected by ecological zone and sowing method, with BS showing significantly higher values than DS and RFS at all three growth stages. At the seedling stage, Ci was significantly affected by ecological zone and sowing method, and Ci in ZY was significantly higher than that in the other two ecological zones. At the regreening stage, ecological zone, cultivar, and sowing method all had significant main effects, and the interaction between ecological zone and cultivar was also significant. BS increased Ci by 3.1% and 1.5% relative to DS and RFS, respectively. Simple effects analysis showed that Ci of L6 was significantly higher than that of L7 in JT and ZY, with increases of 3.8% and 5.6%, respectively. At the flowering stage, Ci was significantly affected by ecological zone and sowing method, and the interaction between ecological zone and cultivar was significant. BS increased Ci by 3.2% and 0.6% relative to DS and RFS, respectively, and Ci of L6 was 5.5% higher than that of L7 in SC.

### 2.5. Effects of Different Sowing Methods on Antioxidant Enzymes in Winter Rapeseed Across Ecological Zones

**Figure 4:**
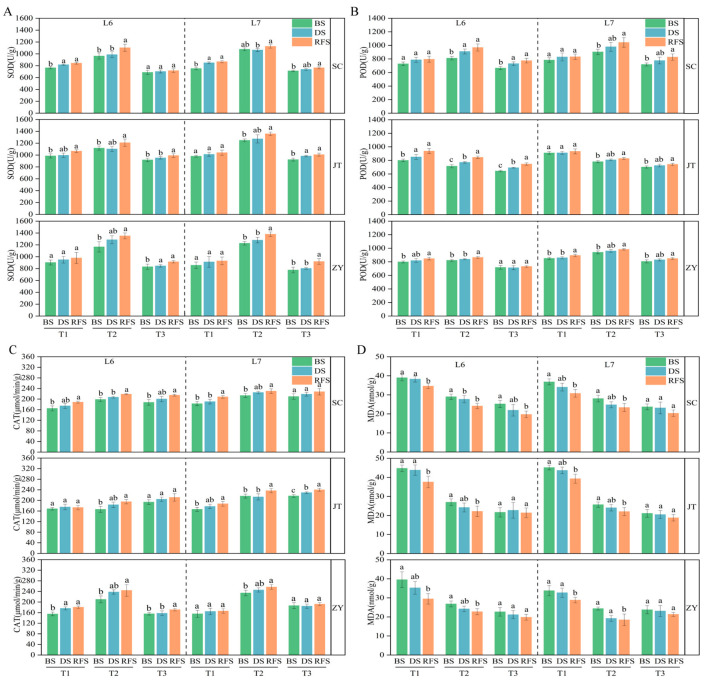
Effects of different sowing methods on antioxidant enzyme activities and malondialdehyde (MDA) content in winter rapeseed leaves at different growth stages across three ecological zones. Panels (**A**–**D**) show superoxide dismutase (SOD) activity, peroxidase (POD) activity, catalase (CAT) activity, and MDA content, respectively. In each panel, the upper, middle, and lower subpanels correspond to the SC, JT, and ZY ecological zones, respectively. Within each subpanel, the sections to the left and right of the dashed line represent Longyou 6 (L6) and Longyou 7 (L7), respectively. T1, seedling stage; T2, regreening stage; T3, flowering stage. BS, broadcast sowing; DS, drill sowing; RFS, ridge–furrow precision sowing. Values are means ± SE (*n* = 3). Different lowercase letters above bars indicate significant differences among sowing methods within the same growth stage, ecological zone, cultivar, and indicator, according to one-way ANOVA followed by Duncan’s multiple range test (*p* < 0.05).

Figure 4 shows the changes in antioxidant enzyme activities and malondialdehyde (MDA) content in winter rapeseed leaves under different sowing methods. Analysis of variance showed that superoxide dismutase (SOD), peroxidase (POD), catalase (CAT), and MDA were significantly affected by ecological zone, cultivar, sowing method, and their interactions, with the effects varying among growth stages and ecological zones.

At the seedling stage, SOD activity was significantly affected by ecological zone and sowing method, whereas the main effect of cultivar was not significant. SOD activity in the JT ecological zone was 24.2% and 9.9% higher than that in the SC and ZY ecological zones, respectively. Among sowing methods, RFS increased SOD activity by 9.2% and 3.4% compared with BS and DS, respectively. At the regreening stage, the interaction between ecological zone and cultivar was significant; SOD activity of L7 was significantly higher than that of L6 in SC and JT, with increases of 7.0% and 13.1%, respectively. At the flowering stage, the main effect of cultivar was significant, with L7 showing 3.4% higher SOD activity than L6. The effect of sowing method on SOD also varied among ecological zones. RFS showed the highest SOD activity in all three ecological zones, and its increase relative to BS followed the order ZY > JT > SC.

POD and CAT showed similar responses at the seedling and regreening stages but differed at the flowering stage. At the seedling stage, both POD and CAT were significantly affected by ecological zone, cultivar, and sowing method, whereas their interaction effects were not significant. Enzyme activities were generally higher in the JT ecological zone. Compared with BS, RFS significantly increased POD and CAT activities by 7.5% and 4.3%, respectively. In addition, POD and CAT activities of L7 were both significantly higher than those of L6, with increases of approximately 6%. At the regreening stage, the interaction between ecological zone and cultivar significantly affected both POD and CAT activities. In SC and JT, POD and CAT activities of L7 were significantly higher than those of L6 by 8.8–14.2% and 7.0–22.2%, respectively. At this stage, sowing method had a significant main effect on CAT activity, with RFS increasing CAT activity by 5.4% compared with BS. In contrast, the effect of sowing method on POD activity depended on ecological zone. In SC, RFS increased POD activity by 17.3% and 6.5% compared with BS and DS, respectively, while the corresponding increases in JT were 11.9% and 5.9%, respectively. In ZY, RFS did not differ significantly from DS, although both treatments showed higher POD activity than BS. At the flowering stage, POD and CAT showed different response patterns. POD activity was significantly affected by the interactions between ecological zone and cultivar and between ecological zone and sowing method. The cultivar effect was mainly observed in ZY, where POD activity of L7 was 14.9% higher than that of L6, whereas no significant cultivar difference was observed in SC or JT. The effect of sowing method on POD also varied among ecological zones. In JT, POD activity followed the order RFS > DS > BS, with RFS being 10.5% and 5.2% higher than BS and DS, respectively. In SC, RFS and DS did not differ significantly, but both were higher than BS, with increases of 15.8% and 9.0%, respectively, relative to BS. No significant difference among sowing methods was observed in ZY. In contrast, CAT activity at the flowering stage was mainly influenced by main effects. CAT activity in JT was 23.7% higher than that in SC, L7 was 12.3% higher than L6, and RFS increased CAT activity by 9.4% and 5.2% compared with BS and DS, respectively.

MDA content was significantly affected by ecological zone and sowing method at all three growth stages, whereas the main effect of cultivar was not significant. The interaction between ecological zone and cultivar was significant at each stage. Across sowing methods, RFS consistently reduced MDA content in winter rapeseed leaves. At the seedling stage, RFS reduced MDA content by 19.2% and 13.6% compared with BS and DS, respectively. Simple effects analysis showed that, in SC, MDA content of L6 was 10.0% higher than that of L7, whereas no significant cultivar difference was observed in JT or ZY. At the regreening stage, RFS reduced MDA content by 21.0% and 8.4% compared with BS and DS, respectively. In ZY, MDA content of L6 was 18.9% higher than that of L7, whereas no significant cultivar difference was observed in SC or JT. At the flowering stage, RFS reduced MDA content by 17.3% and 11.2% compared with BS and DS, respectively. In JT, MDA content of L6 was 9.9% higher than that of L7, whereas no significant cultivar difference was observed in SC or ZY.

Overall, RFS maintained relatively high SOD, POD, and CAT activities and relatively low MDA content across ecological zones and growth stages.

### 2.6. Effects of Sowing Methods on Agronomic Traits and Yields of Winter Rapeseed in Different Ecological Zones

Based on the results of the three-way ANOVA (Table 3), ecological zone (R), variety (V), sowing method (M), and their interactions had significant effects on most agronomic traits and yield of rapeseed. Among these factors, the main effect of sowing method reached significant or highly significant levels for most traits, and significant interactions were also observed with ecological zone and variety. In terms of yield performance, the three sowing methods generally followed the order RFS > DS > BS. Across the three ecological zones, the mean yields of L6 under the BS, DS, and RFS treatments were 2209.9, 2302.8, and 2463.2 kg ha^−1^, respectively, and the average yield increases under RFS relative to BS and DS were 11.46% and 6.97%, respectively. For L7, the mean yields under the BS, DS, and RFS treatments were 2319.1, 2434.6, and 2690.6 kg ha^−1^, respectively, and the average yield increases under RFS relative to BS and DS were 16.02% and 10.52%, respectively. Among the ecological zones, SC had the highest mean yield, reaching 2647.98 kg ha^−1^, followed by JT at 2482.53 kg ha^−1^, whereas ZY was relatively lower at 2079.0 kg ha^−1^. Between varieties, the overall yield of L7 was 2481.00 kg ha^−1^, which was markedly higher than the 2325.3 kg ha^−1^ recorded for L6.

Yield differences were mainly associated with improvements in pod number and related yield components. Across all ecological zones and variety combinations, the total pod number per plant under the RFS treatment was the highest among the three sowing methods, and in most cases it differed significantly from that under BS or DS. For example, in the ZY ecological zone, the total pod number per plant of L6 under the RFS treatment increased by 22.7% and 11.7% compared with the BS and DS treatments, respectively. The number of primary branches also tended to increase under the RFS treatment. In the SC ecological zone, the number of primary branches of L7 under the RFS treatment increased by 2.3–2.6 compared with those under BS and DS, whereas in the JT ecological zone, the corresponding increase was 4.9–5.7. Meanwhile, in some ecological zones, RFS markedly promoted root and stem development, which was conducive to the formation of more effective pods and a more stable plant architecture. The variation in thousand-seed weight among different sowing methods was relatively small, ranging from 2.3 to 2.8 g. However, ANOVA indicated that sowing method and its interactions with ecological zone and variety all had significant effects on thousand-seed weight. In summary, under the experimental conditions of this study, although the responses of different ecological zones and varieties to sowing method varied, ridge–furrow precision sowing generally increased the number of primary branches and the total pod number per plant, thereby improving winter rapeseed yield.

### 2.7. pRDA and Variation Partitioning Analysis of Soil, Physiological, and Yield-Related Traits of Winter Rapeseed Under Different Sowing Methods

**Figure 5:**
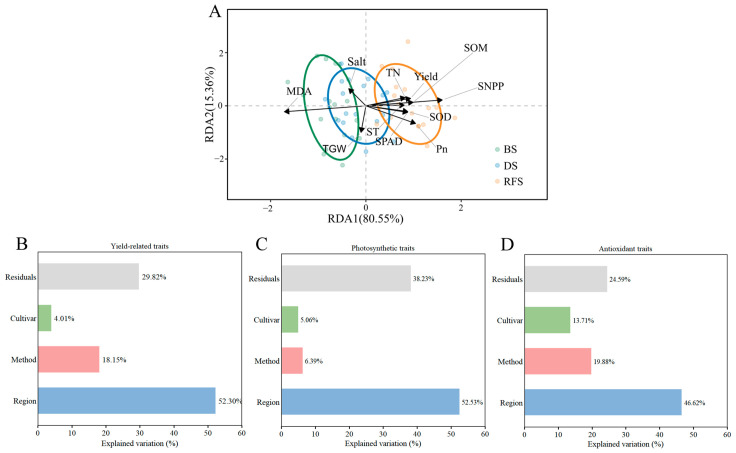
Partial redundancy analysis (pRDA) and variation partitioning of soil, physiological, and yield-related traits of winter rapeseed under different sowing methods. (A) pRDA biplot showing the relationships among sowing methods, soil properties, physiological traits, antioxidant traits, and yield-related traits. Different colored circles represent the BS, DS, and RFS treatments, and ellipses indicate treatment-group distributions. The first two pRDA axes explained 80.55% and 15.36% of the constrained variation, respectively. (B–D) Variation partitioning of yield-related, photosynthetic, and antioxidant traits, respectively, explained by ecological zone, sowing method, cultivar, and unexplained variation. BS, broadcast sowing; DS, drill sowing; RFS, ridge–furrow precision sowing.

Results of the partial redundancy analysis (pRDA) showed that, after controlling for the background effects of ecological zone and cultivar, soil physicochemical factors still significantly explained the variation in physiological and yield-related traits of winter rapeseed (F = 6.9649, *p* = 0.001; Figure 5). The first two ordination axes were both significant, explaining 80.55% and 15.36% of the constrained variation, respectively. Among the environmental factors, salt content, soil organic matter (SOM), nitrogen (N), and soil temperature (ST) all contributed significantly to sample differentiation (*p* < 0.01). The ordination plot showed a clear separation of samples under different sowing methods in the two-dimensional ordination space. Specifically, samples under ridge–furrow precision sowing (RFS) were mainly distributed along the positive direction of pRDA1 and were more closely aligned with the directions of SOM, N, and several physiological and yield-related traits, whereas broadcast sowing (BS) samples were relatively closer to the direction represented by salt content and MDA, and drill sowing (DS) samples were generally intermediate between the two. Among the response variables, the arrows for Yield, SNPP, Pn, SPAD, and SOD were generally aligned with those of SOM and N, but opposite to those of salt content and MDA. In contrast, the ordination direction of thousand-grain weight (TGW) did not completely coincide with that of Yield and SNPP, indicating that its association with the current soil environmental gradient differed from that of the other yield-related traits. Overall, different sowing methods corresponded to differences in soil salt content, nutrient status, and temperature conditions, and were further associated with the physiological performance and yield formation characteristics of winter rapeseed, with RFS samples being overall closer to the direction associated with high SOM, high N, and low salt content.

To further quantify the relative contributions of ecological zone, sowing method, and cultivar to the variation in different trait modules, variation partitioning analysis was conducted. The results showed that, for yield-related traits, the independent explanatory rates of ecological zone, sowing method, and cultivar were 52.30%, 18.15%, and 4.01%, respectively. For photosynthetic traits, the corresponding values were 52.53%, 6.39%, and 5.06%, and for antioxidant traits, they were 46.62%, 19.88%, and 13.71%, respectively. Overall, variation in all three trait modules was explained most strongly by ecological zone, indicating that ecological zone was the primary background factor driving trait variation in winter rapeseed. Meanwhile, the independent explanatory rate of sowing method was relatively high for yield-related and antioxidant traits, whereas the contribution of cultivar to antioxidant traits was also markedly greater than that to yield-related and photosynthetic traits. Overall, although ecological zone dominated the overall variation pattern, sowing method still showed an independent contribution across different trait modules.

## 3. Discussion

### 3.1. The Regulatory Effects of Sowing Methods on the Water–Heat Environment and Physicochemical Properties of Lightly Saline–Alkali Soils

Soil moisture and temperature conditions, together with the physicochemical properties of the tilled soil layer, are widely recognized as key environmental factors regulating root architecture and nutrient uptake efficiency in saline–alkali soils [26,27]. Although ecological zones determine the broader climatic background, the present study showed that sowing methods can further modify the soil microenvironment by reshaping surface microtopography. In particular, ridge–furrow precision sowing (RFS) consistently improved soil moisture and soil temperature across ecological zones. This effect was most evident during the regreening stage, a critical period for post-winter recovery that is highly sensitive to water availability. At this stage, RFS enhanced rainwater collection and moisture conservation within the furrow, resulting in increases of 13.8% and 9.7% in soil moisture and soil temperature, respectively, compared with broadcast sowing in the JT ecological zone. This finding is consistent with previous studies in semiarid agricultural systems showing that ridge–furrow rainwater harvesting cultivation can improve soil moisture and root-zone temperature, thereby promoting biomass accumulation and yield formation under rainfed conditions [28]. Ridge–furrow rainwater harvesting has also been reported to increase water storage in the tilled soil layer and alleviate water deficit [29], while micro-ridge–furrow planting can improve soil water availability and crop water-use efficiency [30]. Therefore, the present results indicate that RFS can improve the hydrothermal regime of saline–alkali soils, although the magnitude of this improvement varies among ecological zones.

On the basis of improved soil moisture and temperature conditions, the interactive responses of soil physicochemical properties to sowing method and ecological zone further reflected environment-dependent nutrient regulation. Significant interactions between ecological zone and sowing method were observed for total carbon (TC), available potassium (AK), and ammonium nitrogen (NH_4_^+^-N), indicating that ridge–furrow microtopography can regulate soil nutrient dynamics differently under different ecological backgrounds. In particular, in the JT ecological zone, RFS was associated with higher TC and NH_4_^+^-N levels, suggesting that improved soil moisture and temperature under ridge–furrow planting may enhance microbial activity, promote organic matter decomposition and nitrogen mineralization, and thereby increase the supply of available nutrients. This interpretation is supported by previous studies showing that ridge–furrow systems can improve soil nutrient dynamics in semiarid agroecosystems by optimizing soil water availability and thermal conditions and by regulating nitrogen transformation and utilization efficiency [31,32]. These improvements in soil hydrothermal and nutrient conditions were closely linked to subsequent physiological performance and yield formation, suggesting that regulation of the tilled-layer soil environment is an important pathway through which RFS promotes winter rapeseed growth.

More importantly, this study extends previous work by evaluating RFS simultaneously across multiple lightly saline–alkali ecological zones and two winter rapeseed cultivars. Rather than only confirming the general water- and temperature-regulating effects of ridge–furrow systems, our results show that the magnitude of these effects depended on ecological background and was further associated with cultivar-specific physiological responses and yield formation. This multi-factor framework provides a more comprehensive basis for matching sowing method with cultivar selection under lightly saline–alkali conditions across different ecological zones.

### 3.2. Effects of Sowing Methods on Photosynthetic Characteristics and Antioxidant Enzyme Physiological Properties in Rapeseed

Soil salinization can reduce root-zone water potential and disrupt ionic homeostasis through osmotic stress and ion toxicity, thereby suppressing stomatal opening and photosynthesis [33,34]. Under saline–alkali stress, crops generally show reduced stomatal conductance (Gs) and net photosynthetic rate (Pn), accompanied by reactive oxygen species (ROS) accumulation and membrane lipid peroxidation. Plants respond to oxidative stress by activating antioxidant enzymes, including superoxide dismutase (SOD), peroxidase (POD), and catalase (CAT), although severe or persistent stress may still cause membrane damage [35,36,37]. Previous studies have shown that ridge planting can improve root-zone moisture conditions and the surrounding microenvironment, thereby enhancing photosynthetic capacity and physiological stress resistance. In winter rapeseed, micro-ridge–furrow sowing has been reported to increase canopy photosynthetically active radiation, Pn, Gs, SPAD values, and chlorophyll fluorescence parameters [38,39]. Similar effects have also been observed in maize and winter wheat, where ridge–furrow planting or mulching increased photosynthetic pigment content and antioxidant enzyme activities while reducing MDA accumulation, thereby improving photosynthetic performance and stress tolerance [40,41]. Consistent with these findings, the present study showed that ridge–furrow precision sowing (RFS) improved the physiological performance of winter rapeseed across ecological zones and growth stages. RFS maintained higher SPAD values and Pn, enhanced Tr more consistently than Gs, and was generally associated with lower Ci than BS or DS. These changes suggest that RFS promoted photosynthetic carbon assimilation rather than merely increasing intercellular CO _2_ accumulation. Meanwhile, RFS increased SOD, POD, and CAT activities and reduced MDA content, indicating that the improved soil moisture, temperature, and nutrient conditions under RFS were associated with stronger antioxidant protection and reduced membrane lipid peroxidation. Therefore, the physiological advantage of RFS reflected a coordinated improvement in photosynthetic activity and oxidative stress defense.

In addition to the general effect of the sowing method, clear cultivar-specific physiological responses were observed, and these responses depended strongly on ecological zone and growth stage. L7 showed higher Pn than L6 in several ecological zone–stage combinations, accompanied by higher Gs or Tr under specific conditions. A similar pattern was observed in the antioxidant system: at the regreening stage, L7 had higher SOD activity than L6 in SC and JT, and its POD and CAT activities were also higher than those of L6 in these two ecological zones. In contrast, MDA content was higher in L6 than in L7 in SC at the seedling stage, in ZY at the regreening stage, and in JT at the flowering stage. These results suggest that L7 may have a stronger capacity to maintain gas exchange, carbon assimilation, and antioxidant defense under lightly saline–alkali field conditions, whereas L6 may be more susceptible to oxidative pressure under certain ecological conditions. The significant interactions between ecological zone and cultivar further indicate that the physiological advantage of L7 was not uniform across all ecological zones, but was concentrated in specific ecological zones and growth stages. This may be related to differences in soil moisture, soil temperature, salinity, and nutrient availability among ecological zones, which altered the relative importance of photosynthetic regulation and antioxidant protection. Therefore, the interaction between ecological zone and cultivar has clear biological significance and suggests that cultivar selection should be matched with local ecological conditions.

Overall, RFS improved the physiological performance of winter rapeseed by simultaneously enhancing photosynthetic activity and antioxidant defense. More importantly, the cultivar-dependent responses observed in this study indicate that the effectiveness of RFS can be further optimized by selecting cultivars with stronger photosynthetic maintenance and antioxidant protection capacity, such as L7, across different ecological zones.

### 3.3. Regulatory Effects of Different Sowing Methods on Winter Rapeseed Yield Formation and Its Constituent Factors

Different cropping systems can affect crop productivity in saline–alkali soils by altering soil water–salt distribution, root growth, canopy development, and yield-component formation. Previous studies have shown that ridge–furrow planting can improve root-zone soil moisture, reduce salinity, and promote crop growth, thereby increasing yield in saline cotton fields and coastal saline–alkali maize fields [42,43]. Ridge–furrow cultivation has also been reported to improve water- and nitrogen-use efficiency by regulating soil water redistribution and nutrient dynamics [30]. These findings suggest that the yield advantage of ridge–furrow systems is closely related to improved soil conditions and their subsequent effects on crop growth and yield-component development. In the present study, ridge–furrow precision sowing (RFS) significantly increased seed yield compared with broadcast sowing (BS) and drill sowing (DS), with average increases of 13.8% and 8.9%, respectively. This yield improvement was associated with improved soil moisture and temperature conditions, enhanced nutrient availability in some ecological zones, and better physiological performance, including higher SPAD values, stronger photosynthetic activity, enhanced antioxidant enzyme activities, and lower MDA accumulation. These changes may have helped winter rapeseed maintain better growth and stress tolerance during key growth stages, especially during post-winter recovery. Among the yield components, increases in the number of primary branches and total siliques per plant were likely important contributors to the yield advantage under RFS. In contrast, thousand-seed weight varied only slightly among sowing methods, suggesting that the yield improvement under RFS was more closely associated with enhanced branching and silique formation than with increases in seed weight. Therefore, RFS may have promoted yield formation by improving vegetative growth, strengthening branching capacity, and increasing silique formation, thereby providing an important agronomic basis for higher seed yield in winter rapeseed.

Cultivar differences also contributed to yield formation. L7 produced higher yield than L6, which was consistent with its stronger physiological performance in several ecological zone–stage combinations, including higher Pn, stronger antioxidant enzyme activities, and lower MDA accumulation under specific conditions. These traits may have allowed L7 to maintain better carbon assimilation and stress protection, thereby supporting more stable reproductive growth under lightly saline–alkali field conditions.

The yield response also depended on ecological zone. Differences in soil moisture, soil temperature, salinity, and nutrient availability among SC, JT, and ZY likely altered the relative importance of different yield-limiting factors. Therefore, the yield advantage of RFS and the stronger performance of L7 should be interpreted as environment-dependent responses. Overall, high-yield formation of winter rapeseed in lightly saline–alkali soils requires the coupling of optimized sowing methods and cultivar adaptability. RFS increased yield mainly by improving soil conditions, maintaining physiological activity, and increasing primary branch number and total silique number per plant, while L7 showed greater potential to convert improved growth conditions into higher yield across ecological zones.

### 3.4. Integrated Interpretation of pRDA and Variation Partitioning

The pRDA and variation partitioning analyses indicated that the beneficial effects of ridge–furrow precision sowing (RFS) on winter rapeseed were unlikely to be explained by improvement in a single trait alone, but rather reflected coordinated changes in soil conditions, physiological performance, antioxidant responses, and yield-related traits. Previous studies have shown that saline–alkali stress disrupts ionic homeostasis, induces ROS accumulation, suppresses photosynthesis, and aggravates lipid peroxidation, whereas reduced salinity and improved nutrient availability are generally associated with better physiological performance and yield formation [44,45].

Consistent with this, samples under RFS were associated with higher SOM and N levels, lower soil salt content, stronger photosynthetic performance and antioxidant capacity, and improved yield-related traits. In contrast, samples under BS were positioned closer to the vectors of soil salt content and MDA, suggesting that higher soil salt content under BS was associated with greater membrane lipid peroxidation and weaker physiological performance. These findings are also consistent with studies showing that ridge–furrow practices can improve soil moisture and temperature regimes and enhance resource-use efficiency [39,46]. Moreover, the distinct ordination direction of thousand-seed weight compared with yield and silique number per plant suggests that the yield advantage under RFS was more closely related to reproductive organ formation than to seed weight alone.

Variation partitioning further showed that ecological zone explained the largest proportion of variation in yield-related, photosynthetic, and antioxidant trait modules, highlighting the dominant role of regional environmental conditions. However, sowing method still explained an independent proportion of variation, particularly for yield-related and antioxidant traits, indicating that the effect of RFS was not fully masked by ecological differences. Overall, this study integrates soil conditions, physiological regulation, antioxidant responses, and yield-related traits within a single analytical framework, suggesting that winter rapeseed production in lightly saline–alkali soils should be optimized by jointly considering sowing method, ecological conditions, and cultivar adaptability.

## 4. Materials and Methods

### 4.1. Study Site Description

This study was conducted from August 2024 to June 2025 across three experimental sites in northwestern China, hereafter referred to as SC, JT, and ZY. The sites were: the rapeseed experimental base in Huang citan Village, Shangchuan Town, Yongdeng County, Gansu Province (36.7° N, 103.6° E); Jiuzhi Village, Jing tai County, Gansu Province (37.8° N, 105.0° E); and Zhang ye City, Gansu Province (37.8° N, 103.6° E). All sites have gray calcareous soils and are classified as lightly saline–alkaline soils. The physicochemical properties of the 0–30 cm soil layer are summarized in Table 4. The preceding crop at all sites was wheat.

### 4.2. Experiment Design and Management

**Figure 6:**
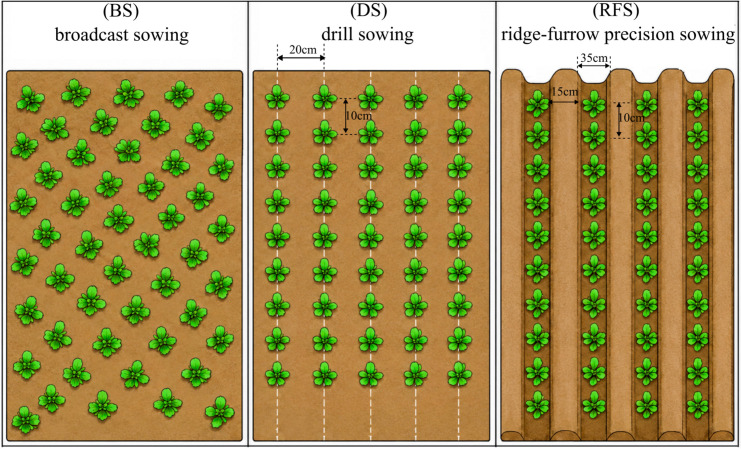
Schematic illustration of the three sowing treatments: broadcast sowing (BS), drill sowing (DS), and ridge–furrow precision sowing (RFS). In the DS treatment, the row spacing and plant spacing were 20 cm and 10 cm, respectively. In the RFS treatment, the furrow width, plant spacing, ridge-top width, and ridge height were 35 cm, 10 cm, 15 cm, and 20 cm, respectively.

The experiment was conducted from August 2024 to June 2025 at three locations in northwestern China (SC, JT, and ZY), and each location was treated as an independent environment. A two-factor split-plot design with three replications was used at each site, arranged in a randomized complete block design. The main plots were assigned to three manual sowing methods: broadcast sowing (BS), drill sowing (DS), and ridge–furrow precision sowing (RFS). The subplots were assigned to two strong winter rapeseed (*Brassica rapa* L.) cultivars, Longyou 6 (L6) and Longyou 7 (L7), both developed by Gansu Agricultural University. Their main agronomic and morphological traits are presented in Table 5. The experimental fields consisted of lightly saline–alkaline soils, with wheat as the preceding crop. Residues of the previous wheat crop were incorporated into the soil by plowing. Three sowing treatments were used: broadcast sowing (BS), drill sowing (DS), and ridge–furrow sowing (RFS). In the BS treatment, seeds were evenly broadcast by hand on the soil surface, followed by light harrowing and manual rolling to improve seed–soil contact. In the DS treatment, seeds were sown in manually opened 10 cm-deep furrows at a depth of 3–5 cm, with 10 cm between plants and 20 cm between rows, and were covered with 1–2 cm of soil without rolling. In the RFS treatment, ridges with a height of 20 cm and a top width of 15 cm were manually formed, with 35 cm-wide furrows between ridges. Seeds were sown at the furrow bottom at a depth of 3–5 cm, with 10 cm between plants and 35 cm between rows, and were covered with 1–2 cm of soil without rolling. The schematic diagram of the BS, DS, and RFS treatments is presented in Figure 6. The sowing rates for BS, DS, and RFS were 6.5, 6.0, and 6.0 kg ha^−1^, respectively. Basal fertilization consisted of 255 kg ha^−1^ diammonium phosphate (18% N and 46% P_2_O_5_) and 105 kg ha^−1^ urea (46% N), corresponding to 94.2 kg N ha^−1^ and 51.1 kg P ha^−1^ as pure nutrients, equivalent to 117.3 kg P_2_O_5_ ha^−1^. No potassium fertilizer was applied, corresponding to 0 kg K ha^−1^. At the regreening stage, an additional 46.0 kg N ha^−1^ was applied as urea. Therefore, the total nutrient input during the growing season was 140.2 kg N ha^−1^, 51.1 kg P ha^−1^, and 0 kg K ha^−1^.

To ensure uniform emergence and reduce soil salinity in the plow layer, pre-sowing leaching irrigation was applied 15–20 days before sowing (2700–3000 m^3^ ha^−1^, equivalent to 270–300 mm). During the growing season, supplemental irrigation was applied at three critical stages: before winter dormancy (900 m^3^ ha^−1^, 90 mm), at the regreening stage (450 m^3^ ha^−1^, 45 mm), and at full flowering (450 m^3^ ha^−1^, 45 mm). The total supplemental irrigation during the growing season, excluding pre-sowing irrigation, was approximately 1800 m^3^ ha^−1^ (180 mm). Irrigation was performed using surface (flood) irrigation, and all treatments were managed identically across the three sites. Pest and disease management followed local high-yield cultivation practices. Each main plot measured 48 m^2^, and each subplot 24 m^2^ (3 m × 8 m), with guard rows surrounding both main and subplots.

### 4.3. Data Collection

#### 4.3.1. Records of the Fertile Period

We recorded the timing when each winter rapeseed treatment entered its critical growth stage, based on the date when 70% of plants within the plot reached that stage. We also calculated the overwintering rate.

Overwintering rate (%) = (number of plants surviving after overwintering/total number of plants before overwintering) × 100%.

#### 4.3.2. Photosynthetic Index Measurement

During the seedling, regreening, and flowering stages of winter rapeseed, leaf photosynthetic parameters were measured using a Li-6400XT photosynthesis system (Li-Cor, Lincoln, NE, USA). Measurements were conducted daily between 09:00 and 11:30, and the most recent fully expanded functional leaf on the main stem of each plant was selected. Measured parameters included net photosynthetic rate (Pn), intercellular CO_2_ concentration (Ci), stomatal conductance (Gs), and transpiration rate (Tr). Additionally, SPAD values were determined using a portable chlorophyll meter (TYS-B, Zhejiang Top Instrument Co., Ltd., Hangzhou, China) on leaves from the same position to represent relative chlorophyll content. Three plants per subplot were randomly selected for these measurements.

#### 4.3.3. Antioxidant Enzyme Assay

Leaves were collected at the seedling, regreening, and flowering stages for determination of antioxidant enzyme activities and malondialdehyde (MDA) content. Samples were immediately frozen in liquid nitrogen, transported to the laboratory, and stored at −80 °C until analysis. Activities of superoxide dismutase (SOD), peroxidase (POD), and catalase (CAT), as well as MDA content, were determined using commercial assay kits (Ruixin Biotechnology Co., Ltd., Quanzhou, China) following the manufacturer’s instructions. Three plants per subplot were randomly selected for these measurements.

#### 4.3.4. Soil Sampling and Determination of Relevant Parameters

Before sowing, at the regreening stage, and after harvest, soil samples were collected from the 0–30 cm soil layer of each subplot using the five-point sampling method. In each subplot, five sampling points were selected in a quincunx pattern, with one point near the center and four points near the corners while avoiding plot borders. One soil core was collected from each sampling point, and the five soil cores from the same subplot were thoroughly mixed to form one composite sample [47]. For the analysis of soil physicochemical properties, a portion of each composite sample was air-dried, ground, and passed through a 2 mm sieve. Measured properties included pH, salinity, organic matter, total nitrogen, total phosphorus, total potassium, total carbon, available phosphorus, and available potassium. Another portion of each composite sample was kept as a fresh subsample at 4 °C for nitrate (NO_3_^−^-N) and ammonium (NH_4_^+^-N) analysis. Measurement methods followed established protocols from previous studies [48,49]. Soil temperature and soil moisture at 0–30 cm depth were measured in the field using a portable soil water and temperature sensor (Top Yunong TZS-ECWY-6G; Top Yunong Technology Co., Ltd., Hangzhou, China).

#### 4.3.5. Measurement of Agronomic Traits and Yield Components

At maturity, five representative plants were randomly selected from each plot for yield component analysis. Yield-related traits, including root length, root diameter, stem diameter, effective branch number per plant, pod number per plant, seed number per pod, and 1000-seed weight, were measured. In each plot, a 5 m^2^ area was harvested for yield determination. After threshing, the seeds were naturally air-dried and weighed, and seed yield was converted to a unit-area basis (kg ha^−1^). Yield and yield component measurements were conducted according to the method described by Li et al. [50].

### 4.4. Statistical Analysis

A three-way analysis of variance (ANOVA) was performed to evaluate the main and interactive effects of ecological zone, cultivar, and sowing method on soil physicochemical properties, photosynthetic characteristics, antioxidant-related physiological traits, yield, and yield components of winter rapeseed. Because some measurements were collected at different growth stages, the ANOVA was conducted separately for each growth stage where appropriate. When the effects were significant, mean comparisons were performed using Duncan’s multiple range test at the 0.05 probability level. Differences were considered significant at *p* < 0.05. All statistical analyses were performed using SPSS 20.0 (SPSS Inc., Chicago, IL, USA), and figures were prepared using Origin 2021 (OriginLab Corp., Northampton, MA, USA).

Partial redundancy analysis (pRDA) was used to examine the relationships between soil physicochemical factors and winter rapeseed physiological and yield-related traits. The response matrix included superoxide dismutase activity (SOD), malondialdehyde content (MDA), SPAD value, net photosynthetic rate (Pn), seed number per pod (SNPP), 1000-seed weight (TGW), and seed yield. Soil salinity, soil organic matter (SOM), total nitrogen (TN), and soil temperature (ST) were used as explanatory soil variables, while ecological zone and cultivar were included as conditional variables to control for background effects.

Variance partitioning was used to quantify the relative contributions of ecological zone, sowing method, and cultivar to the variation in yield-related traits, photosynthetic traits, and antioxidant traits. All analyses were conducted in R (version 4.3.1) using the vegan package for pRDA, variance partitioning, and permutation tests. The significance of the pRDA model, constrained axes, and explanatory soil variables was assessed based on 999 permutations. Ordination plots and variance partitioning plots were generated using the ggplot2, ggrepel, and grid packages.

## 5. Conclusions

Based on field experiments across three ecological zones, this study tested the hypothesis that ridge–furrow precision sowing (RFS) would improve the root-zone soil environment, enhance physiological performance and antioxidant defense, and promote yield formation of winter rapeseed under lightly saline–alkali conditions, with responses differing between cultivars and ecological zones. The results generally supported this hypothesis. Compared with broadcast sowing (BS) and drill sowing (DS), RFS improved soil moisture and temperature conditions, maintained higher SPAD values and photosynthetic performance, enhanced antioxidant enzyme activities, and reduced MDA accumulation. These changes were associated with increases in primary branch number and total silique number per plant, resulting in seed yield increases of 13.8% and 8.9% compared with BS and DS, respectively.

Ecological zone was the dominant factor shaping variation in soil, physiological, antioxidant, and yield-related traits, indicating that regional environmental conditions strongly influenced winter rapeseed performance. However, the sowing method still made an independent contribution, particularly to yield-related and antioxidant traits, suggesting that the effect of RFS was not fully masked by ecological differences. Cultivar differences were also evident. L7 generally showed higher yield than L6 and exhibited stronger photosynthetic performance and antioxidant defense under specific ecological conditions, whereas L6 showed higher MDA or Ci in some ecological zone–stage combinations. These findings indicate that cultivar adaptability is an important factor affecting the response of winter rapeseed to lightly saline–alkali environments and sowing methods.

Overall, RFS is an effective sowing method for improving winter rapeseed productivity in saline–alkali soils, mainly by improving soil hydrothermal conditions, strengthening physiological stress tolerance, and promoting yield-component formation. However, the response to RFS depends on ecological zone and cultivar. Therefore, optimizing winter rapeseed production in lightly saline–alkali areas should combine suitable sowing methods with cultivar selection adapted to local ecological conditions, with L7 showing greater potential than L6 under the conditions of this study.

## Figures and Tables

**Table 1:** Phenological stages and overwintering rates of winter rapeseed in different ecological zones under various sowing methods.

Region	Treatment	Sowing Period (D/M)	Emergence Period (D/M)	Regreening Period (D/M)	Budding Period (D/M)	Flowering Period (D/M)	Maturity Period (D/M)	Growth Period (Day)	Overwintering Rate (%)
SC	L6BS	16/8	21/8	16/4	23/4	5/5	21/6	310	80.4
	L6DS	16/8	21/8	15/4	24/4	4/5	19/6	308	83.2
	L6RFS	16/8	22/8	13/4	22/4	2/5	16/6	305	84.5
	L7BS	16/8	21/8	17/4	24/4	7/5	21/6	310	83.3
	L7DS	16/8	22/8	16/4	24/4	6/5	21/6	310	82.5
	L7RFS	16/8	23/8	14/4	22/4	6/5	19/6	308	85.6
JT	L6BS	21/8	26/8	16/4	25/4	8/5	13/6	305	76.3
	L6DS	21/8	26/8	15/4	27/4	9/5	14/6	306	80.5
	L6RFS	21/8	27/8	14/4	27/4	9/5	16/6	308	83.5
	L7BS	21/8	26/8	14/4	25/4	7/5	12/6	304	75.5
	L7DS	21/8	26/8	15/4	26/4	9/5	14/6	306	79.6
	L7RFS	21/8	26/8	15/4	26/4	8/5	15/6	307	83.2
ZY	L6BS	15/8	21/8	18/4	23/4	2/5	13/6	302	78.5
	L6DS	15/8	21/8	18/4	24/4	4/5	14/6	303	80.3
	L6RFS	15/8	21/8	16/4	22/4	4/5	14/6	303	82.4
	L7BS	15/8	21/8	17/4	24/4	5/5	16/6	305	76.5
	L7DS	15/8	21/8	17/4	24/4	3/5	16/6	304	78.3
	L7RFS	15/8	22/8	16/4	22/4	4/5	13/6	302	82.2

Note: BS, broadcast sowing; DS, drill sowing; RFS, ridge–furrow precision sowing. SC, JT, and ZY denote the three ecological zones. L6 and L7 denote the two winter rapeseed cultivars.

**Table 2:** Effects of different sowing methods on soil physicochemical properties at the regreening stage of winter rapeseed across ecological zones.

R	V	M	TN (mg kg^−1^)	TP (g kg^−1^)	TK (g kg^−1^)	TC (mg kg^−1^)	pH	Salt (%)	AK (mg kg^−1^)	AP (mg kg^−1^)	SOM (g kg^−1^)	NO_3_^-^-N (mg kg^−1^)	NH_4_^+^-N (mg kg^−1^)
SC	L6	BS	0.503 a	0.870 a	21.57 a	11.48 a	8.47 ab	0.087 a	190.33 c	17.25 a	11.90 a	22.61 b	8.64 a
		DS	0.448 a	0.903 a	21.80 a	11.48 a	8.52 a	0.073 b	223.00 c	19.43 a	12.57 a	25.31 ab	8.90 a
		RFS	0.488 a	0.883 a	20.60 a	11.61 a	8.44 b	0.067 b	233.33 a	19.64 a	12.87 a	27.95 a	9.70 a
	L7	BS	0.539 a	0.913 a	21.35 a	11.52 a	8.47 a	0.080 a	206.00 b	22.61 a	11.93 b	23.41 b	9.36 c
		DS	0.51 a	0.870 a	21.13 a	11.34 a	8.42 ab	0.070 a	230.67 a	20.74 a	12.83 ab	26.23 ab	10.66 b
		RFS	0.548 a	0.917 a	21.77 a	10.21 a	8.37 b	0.063 a	233.33 a	21.91 a	13.87 a	27.83 a	12.05 a
JT	L6	BS	0.507 b	0.843 a	19.57 ab	11.00 b	8.32 a	0.220 a	332.67 b	9.29 a	13.87 b	11.22 b	3.38 a
		DS	0.556 a	0.847 a	20.07 a	10.84 b	8.31 a	0.210 a	337.00 ab	8.30 a	14.95 ab	15.27 a	3.60 a
		RFS	0.552 a	0.850 a	19.07 b	11.62 a	8.35 a	0.200 a	347.33 a	8.01 a	14.69 a	15.81 a	4.03 a
	L7	BS	0.537 b	0.827 a	19.80 a	11.61 a	8.46 a	0.163 a	328.00 a	14.61 ab	13.70 b	13.35 a	3.73 a
		DS	0.548 b	0.867 a	20.30 a	11.20 b	8.45 a	0.157 a	330.33 a	13.26 b	14.17 ab	16.49 a	3.81 a
		RFS	0.589 a	0.843 a	20.03 a	11.83 a	8.51 a	0.150 a	336.67 a	17.23 a	14.83 a	17.00 a	4.22 a
ZY	L6	BS	0.442 b	0.820 b	15.70 a	16.24 b	8.51 a	0.267 a	173.33 c	14.74 a	11.40 b	48.37 a	3.68 a
		DS	0.448 b	0.817 b	16.00 a	16.02 b	8.46 b	0.257 a	187.00 b	15.38 a	12.73 ab	54.65 a	4.36 a
		RFS	0.519 a	0.857 a	14.47 b	17.33 a	8.43 b	0.197 b	192.67 a	17.19 a	13.47 a	53.40 a	4.74 a
	L7	BS	0.467 b	0.817 b	16.23 a	14.64 b	8.57 a	0.170 a	194.67 a	14.84 a	11.13 b	49.99 a	3.60 b
		DS	0.472 ab	0.870 a	16.23 a	15.24 a	8.60 a	0.163 ab	211.67 a	16.11 a	12.60 a	50.48 a	3.68 b
		RFS	0.49 a	0.877 a	15.63 a	15.18 a	8.58 a	0.147 b	213.00 a	16.44 a	12.83 a	48.62 a	5.18 a
ANOVA													
R			25.10 **	11.34 *	294.78 **	590.92 **	52.95 **	418.58 **	4726.13 **	52.31 **	40.12 **	1283.40 **	860.83 **
V			9.23 **	1.63 ns	4.26 *	19.39 **	41.44 **	139.78 **	233.30 **	21.90 **	0.09 ns	0.05 ns	18.83 **
M			6.35 **	1.91 ns	3.92 *	1.85 ns	13.86 **	16.88 **	324.05 **	1.38 ns	18.51 **	14.81 **	30.23 **
R×V			2.51 ns	0.56 ns	0.69 ns	20.27 **	38.62 **	32.44 **	253.54 **	7.63 **	1.57 ns	3.98*	15.16 **
R×M			2.39 ns	0.85 ns	0.75 ns	5.09 **	0.48 ns	2.97 *	85.09 **	0.33 ns	0.74 ns	1.27 ns	2.71 *
V×M			0.06 ns	0.06 ns	3.26 ns	5.63 **	0.01 ns	2.25 ns	153.94 ns	0.38 ns	0.35 ns	1.99 ns	2.39 ns
R×V×M			1.25 ns	1.73 ns	0.27 ns	1.41 ns	0.58 ns	1.43 ns	228.27 ns	1.10 ns	0.60 ns	1.05 ns	1.90 ns

Note: Values are means ± SE (*n* = 3). Different lowercase letters indicate significant differences among sowing methods within the same ecological zone and cultivar for each variable, according to Duncan’s multiple range test (*p* < 0.05). * and ** indicate significance at *p* < 0.05 and *p* < 0.01, respectively; ns indicates not significant. BS, broadcast sowing; DS, drill sowing; RFS, ridge–furrow precision sowing. R, ecological zone; V, cultivar; M, sowing method.

**Table 3:** Effects of different sowing methods on agronomic traits and yields of winter rapeseed across ecological zones.

R	V	M	Plant Height (cm)	Root Length (cm)	Root Diameter (cm)	Stem Diameter (cm)	Branching Height (cm)	Primary Branches (number)	Total Pods per Plant (number)	Thousand-Seed Weight (g)	Yield (kg ha^−1^)
SC	L6	BS	96.38 b	14.16 b	1.58 a	0.94 a	5.44 b	7.8 b	139.8 b	2.81 a	2386.2 b
		DS	102.5 ab	14.96 ab	2.02 a	1.02 a	10.98 a	8.8 ab	146.4 b	2.77 a	2524.0 ab
		RFS	113.32 a	17.56 a	2.04 a	1.06 a	13.24 a	9.8 a	169.8 a	2.76 a	2634.5 a
	L7	BS	96.46 b	13.04 a	1.34 a	0.60 b	8.32 a	6.8 b	154.8 b	2.71 a	2632.4 b
		DS	108.12 ab	11.88 a	1.3 a	0.62 b	15.42 a	7.1 b	157.8 b	2.68 a	2764.7 ab
		RFS	108.86 a	15.112 a	1.39 a	0.76 a	11.6 a	9.4 a	173.0 a	2.65 a	2946.2 a
JT	L6	BS	103.92 b	11.78 b	1.20 b	0.64 a	21.28 a	7.8 a	134.6 a	2.58 a	2357.75 b
		DS	111.56 ab	14.03 ab	1.48 ab	0.64 a	21.76 a	8.8 a	136.0 a	2.56 a	2373.15 b
		RFS	115.12 a	15.46 a	1.8 a	0.8 a	17.18 a	9.8 a	154.8 a	2.59 a	2693.75 a
	L7	BS	99.12 a	12.52 b	1.34 a	0.51 b	13.12 b	8.4 b	139.4 b	2.65 a	2263.6 b
		DS	98.54 a	15.2 a	1.46 a	0.69 b	7.4 c	7.6 b	148.2 b	2.59 a	2401 a
		RFS	108.72 a	16.78 a	1.574 a	0.94 a	22.68 a	13.34 a	184.0 a	2.60 a	2806 a
ZY	L6	BS	70.7 a	13.16 a	1.06 b	0.51 a	8.22 a	7 a	157.2 c	2.60 a	1885.85 b
		DS	77.08 a	12.94 a	1.16 b	0.51 a	5.24 ab	7.8 a	177.0 b	2.64 a	2011.4 ab
		RFS	74.88 a	17.5 a	1.94 a	0.62 a	3.7 c	8.6 a	197.4 a	2.66 a	2061.4 a
	L7	BS	73.48 a	16.76 a	1.42 a	0.58 a	2.94 a	9.4 a	165.6 b	2.65 a	2057.5 b
		DS	70.68 a	14.94 a	0.96 a	0.50 a	4.26 a	7.8 a	181.8 b	2.68 a	2138.1 ab
		RFS	79.68 a	16.32 a	1.58 a	0.6 a	5.4 a	8 a	203.2 a	2.65 a	2319.6 a
ANOVA											
R			56.58 **	0.84 **	8.44 ns	16.28 **	40.91 ns	2.38 **	55.74 **	50.19 **	158.26 **
V			1.02 ns	1.10 ns	21.11 ns	4.52 **	2.15 ns	0.03 **	17.87 **	1.34 ns	33.59 **
M			4.57 *	4.66 ns	8.31 **	8.17 **	1.70 *	5.59 **	57.42 **	0.61 ns	46.91 **
R×V			1.56 ns	2.13 **	9.35 ns	16.28 **	6.24 ns	1.25 **	1.12 ns	15.18 **	7.58 **
R×M			1.38 ns	1.18 ns	0.98 *	1.46 ns	1.57 ns	2.73 ns	1.55 ns	2.77 **	3.28 **
V×M			0.33 ns	1.17 *	2.48 ns	0.82 ns	4.93 ns	0.83 ns	0.20 ns	1.21 ns	1.85 ns
R×V×M			1.36 ns	1.11 **	0.53 ns	5.38 ns	4.28 ns	1.68 ns	1.66 ns	0.30 ns	0.37 ns

Note: Different lowercase letters within the same column indicate significant differences among sowing methods within the same ecological zone and variety (*p* < 0.05). * and ** indicate significant differences at the 0.05 and 0.01 levels, respectively; ns indicates no significant difference. BS, broadcast sowing; DS, drill sowing; RFS, ridge–furrow precision sowing. R, ecological zone; V, variety; M, sowing method.

**Table 4:** Chemical properties of soil at different experimental sites (0–30 cm depth).

Region	TN (mg kg^−1^)	TP (g kg^−1^)	TK (g kg^−1^)	AK (mg kg^−1^)	AP (mg kg^−1^)	pH	Salt (%)	NO_3_^-^-N (mg kg^−1^)	NH_4_^+^-N (mg kg^−1^)
SC	0.50	0.88	21	209.5	17.58	8.49	0.09	11.52	8.9
JT	0.57	0.89	19.98	329.1	9.29	8.43	0.15	12.13	7.23
ZY	0.44	0.75	15.91	183.58	10.07	8.51	0.18	14.02	11.85

**Table 5:** Agronomic traits, cold tolerance, and seed quality characteristics of the two winter rapeseed cultivars.

Cultivar	GP (d)	CT	OR (%)	Plant Height (cm)	BNP	SNMP	SNPP	SPS	TSW (g)	YPP (g)	Oil (%)	EA (%)	GSL (μmol g^−1^)
L6	288–295	Extremely strong; −32.0 °C	80–95	107.2	10	54.8	240.6	21.1	3.0	14.1	43.3	44.3	31.9
L7	288–295	Strong; −31.9 °C	≥80	113.5	14	49.8	295.6	20.6	3.1	13.5	43.72	46.99	17.6

Note: GP, growth period; CT, cold tolerance; OR, overwintering rate; BNP, branches per plant; SNMP, number of siliques on the main inflorescence; SNPP, number of siliques per plant; SPS, seeds per silique; TSW, thousand-seed weight; YPP, yield per plant; EA, erucic acid; GSL, glucosinolate. CT was characterized based on low-temperature tolerance and overwintering rate.

## Data Availability

The original contributions presented in this study are included in the article. Further inquiries can be directed to the corresponding author.
